# Saposin C Coupled Lipid Nanovesicles Specifically Target Arthritic Mouse Joints for Optical Imaging of Disease Severity

**DOI:** 10.1371/journal.pone.0033966

**Published:** 2012-03-28

**Authors:** Xiaoyang Qi, Matthew J. Flick, Malinda Frederick, Zhengtao Chu, Rachel Mason, Monica DeLay, Sherry Thornton

**Affiliations:** 1 Division of Hematology-Oncology, Departments of Internal Medicine and Pediatrics, University of Cincinnati College of Medicine, Cincinnati Children's Hospital Medical Center, Cincinnati, Ohio, United States of America; 2 Division of Human Genetics, Departments of Internal Medicine and Pediatrics, University of Cincinnati College of Medicine, Cincinnati Children's Hospital Medical Center, Cincinnati, Ohio, United States of America; 3 Division of Experimental Hematology, Departments of Internal Medicine and Pediatrics, University of Cincinnati College of Medicine, Cincinnati Children's Hospital Medical Center, Cincinnati, Ohio, United States of America; 4 Division of Rheumatology, Departments of Internal Medicine and Pediatrics, University of Cincinnati College of Medicine, Cincinnati Children's Hospital Medical Center, Cincinnati, Ohio, United States of America; University of Southern California, United States of America

## Abstract

Rheumatoid arthritis is a chronic inflammatory disease affecting approximately 1% of the population and is characterized by cartilage and bone destruction ultimately leading to loss of joint function. Early detection and intervention of disease provides the best hope for successful treatment and preservation of joint mobility and function. Reliable and non-invasive techniques that accurately measure arthritic disease onset and progression are lacking. We recently developed a novel agent, SapC-DOPS, which is composed of the membrane-associated lysosomal protein saposin C (SapC) incorporated into 1,2-dioleoyl-*sn*-glycero-3-phospho-L-serine (DOPS) lipid nanovesicles. SapC-DOPS has a high fusogenic affinity for phosphatidylserine-enriched microdomains on surfaces of target cell membranes. Incorporation of a far-red fluorophore, CellVue Maroon (CVM), into the nanovesicles allows for *in vivo* non-invasive visualization of the agent in targeted tissue. Given that phosphatidylserine is present only on the inner leaflet of healthy plasma membranes but is “flipped” to the outer leaflet upon cell damage, we hypothesized that SapC-DOPS would target tissue damage associated with inflammatory arthritis due to local surface-exposure of phosphatidylserine. Optical imaging with SapC-DOPS-CVM in two distinct models of arthritis, serum-transfer arthritis (e.g., K/BxN) and collagen-induced arthritis (CIA) revealed robust SapC-DOPS-CVM specific localization to arthritic paws and joints in live animals. Importantly, intensity of localized fluorescent signal correlated with macroscopic arthritic disease severity and increased with disease progression. Flow cytometry of cells extracted from arthritic joints demonstrated that SapC-DOPS-CVM localized to an average of 7–8% of total joint cells and primarily to CD11b+Gr-1+ cells. Results from the current studies strongly support the application of SapC-DOPS-CVM for advanced clinical and research applications including: detecting early arthritis onset, assessing disease progression real-time in live subjects, and providing novel information regarding cell types that may mediate arthritis progression within joints.

## Introduction

Rheumatoid arthritis (RA) is a chronic inflammatory disease that affects 1% of the population. RA is manifested as chronic inflammation leading to severe pain and loss of mobility. If unchecked, disease culminates in destruction of joint tissue, loss of function, and a profound reduction in quality of life. Several effective biologics have been developed over the last decade that limit arthritis progression for a large percentage of RA patients, and in a number of cases, achieved remission. These therapies are most effective when used to intervene early in the RA disease process; however, reliable and manageable tools for evaluation of early joint inflammation and disease remission in response to therapeutics are lacking. Recently, new guidelines put forth by the American College of Rheumatology (ACR) and The European League Against Rheumatism (EULAR) outlined standard assessments of measuring clinical outcomes, which are needed both for clinical practice and clinical trials [1]. These proposed definitions of RA remission do not include imaging results but do indicate an association with slowing of radiographic progression as residual synovitis may exist in many patients whose disease appears inactive based on conventional clinical evaluation. While radiographic progression can assess joint damage, soft tissue inflammation and destruction are not readily detected by radiography. A consensus has not been established regarding assessment of arthritic events involving inflammatory soft tissue with regards to imaging methods due in part, to the absence of a reliable, quantitative methodology.

Joint counts are part of the ACR and EULAR proposed criteria, and an essential part of the clinical component of assessing rheumatic disease. However, several studies have suggested that differences in joint counts do not necessarily correlate with clinical outcomes (reviewed in [2]). Thus, an objective and quantifiable clinical parameter that correlates with joint damage and inflammation would be highly valuable to the clinic, and imaging has been proposed as a means to provide this missing vital information. Several imaging methods are currently in use for the assessment of joint damage, including X-ray, magnetic resonance imaging (MRI), ultrasound, and X-ray computed tomography [3]–[6]. For clinical ease, X-rays have been the standard for assessment of joint damage progression, which is estimated by joint space narrowing and erosions [7]. Multiple quantitative assessment methods for evaluating radiographic damage have been proposed [6]. While bone structures are readily visible by x-ray, soft tissue inflammation and destruction, which are associated with early stages of RA, are not readily detected. Both magnetic resonance imaging (MRI) and ultrasound have been looked to for assessment of early joint damage. MRI can provide superior imaging of soft tissues and edema, as compared to conventional x-ray, but is time-consuming, expensive and not readily available to many clinicians. Ultrasound can also provide information regarding soft tissue damage and cartilage and bone destruction; however, while ultrasound may be easier to access in the clinic than MRI, training of personnel, reproducibility and joint accessibility remain as significant challenges for bringing this imaging modality to the clinic.

Optical imaging is a relatively new imaging modality and provides great promise for analyzing events occurring early in the pathogenesis of arthritis (reviewed in [8], [9]). Optical imaging uses far-red and near-infrared (600–1000 nm range) [10], [11] molecular or nanoparticle probes that when excited emit light that can penetrate the skin more readily than probes with lower emission wavelengths, facilitating signal detection *in vivo*. Advantages of optical imaging include (1) the decrease in exposure to radiation as compared to X-ray (2) the clear targeting and localization of molecular probes associated with disease pathogenesis and (3) minimal acquisition time.

Targeting of damaged tissue to enhance imaging of inflammation in arthritis could provide a valuable tool for the early assessment of disease and for the progression of disease. In the current study, we have characterized the targeting of novel nanovesicles composed of 1,2-Dioleoyl-*sn*-Glycero-3-Phospho-L-Serine (DOPS) and a fusogenic protein, saposin C (SapC) to image arthritic joints. SapC associates with phosphatidylserine (PS) on cell membranes by embedding amino and carboxyl-end amphipathic helices into the outer leaflet of membranes [12]. PS becomes exposed on the outer membrane of cells that are damaged or undergoing apoptosis. Since SapC-DOPS engages cell membranes with exposed PS, we hypothesized that SapC-DOPS may localize to cells involved in inflammation/injury in arthritis.

The current study determines the signal distribution in mice of SapC-DOPS labeled with a far-red fluorophore, CellVue® Maroon (CVM), as assessed by optical imaging in two arthritis mouse models that each exhibit different disease courses and etiology. Furthermore, the intensity of SapC-DOPS signal as compared with arthritic severity over the course of disease was determined and cell types within joints that are targeted by SapC-DOPS were investigated. Our results suggest that SapC-DOPS localizes specifically to cells involved in early inflammatory stages of arthritis and that this novel agent has potential use as an imaging agent for inflammatory arthritis.

## Materials and Methods

### Animal Use

#### Ethics statement

All experiments involving mice complied with National Institutes of Health guidelines. The protocol was approved by the Institutional Animal Care and Use Committee of the Cincinnati Children's Hospital Research Foundation (Animal Welfare Assurance Number A3108-01).

### Collagen-induced arthritis (CIA)

DBA/1J male mice eight weeks of age were injected intradermally at the base of the tail with 100 µg of bovine type II collagen (CII) (Elastin Products Co., Inc.) in complete Freund's adjuvant (CFA) on day 1 and given a similar challenge once again 21 days following the initial CII injection, as previously described [13], [14]. Mice were evaluated for macroscopic arthritis using an arthritic index macroscopic scoring system ranging from 0 to 4 (0 = no detectable arthritis, 1 = swelling and/or redness of paw or one digit, 2 = two joints involved, 3 = three joints involved and 4 = severe arthritis of the entire paw and digit).

### K/BxN arthritis

Sera were collected from KRN×NOD F1 mice, and 150 µl was given by intraperitoneal injection to C57Bl/6J mice at day 0 of disease. Calipers were used to measure paw thickness and mouse ankle thickness. For ankle thickness, elliptical area of mouse ankles was calculated using intermalleolar measurements and measurement of the area between the dorsal talus and calcaneus.

### IVIS® live imaging of SapC-DOPS-CVM

An aliquot of CellVue® Maroon (CVM, Molecular Targeting Technologies Inc., West Chester, PA) in ethanol was mixed with phospholipid solvent for bath sonication preparation by the procedure as previously described [15], [16]. CVM-labeled SapC-DOPS nanovesicles were separated from free CVM dye using a Sephadex™ G25 column (PD-10, Amersham Pharmacia Biotech, Piscataway, NJ). CVM-labeled SapC-DOPS (SapC = 4.2 mg/kg, DOPS = 2 mg/kg, CVM = 6 µmol) and control agents were administered i.p. or by tail vein injection in a 200 microliter volume into animals. Real-time live images were taken using an IVIS 200 Series imaging system with an XFO-6 fluorescent kit and quantified using Living Image software (Xenogen, Alameda, CA). Average radiance values (IVIS® intensity) of designated elliptical areas of the front and hind paws were determined and the mean values between groups compared.

### Flow Cytometry

For joint cell analysis, mice were sacrificed, paws were removed above the ankle joint, skin was dissected away and the remaining tissue was placed in 0.5 ml of RPMI. Using a number 10 scalpel blade and forceps, tissue was separated from bones and joints and dissected/shredded as previously described [17]. Crude cell suspensions were strained through a 70 micron cell strainer and cells counted. 1×10^6^ cells were stained in 100 ul of FACS buffer (1XPBS, 0.2%BSA) containing 10 ul of 2.4G2 (American Type Culture Collection) hybridoma supernatant. Three antibody panels were assessed for cells from CIA treated mice as follows: panel 1 = anti-CD4-FITC (clone GK1.5, BD Pharmingen, San Jose, CA), anti-CD8-Pacific Blue (clone 53-6.7, BD Pharmingen,) and anti-CD19-PE (clone ID3, BD Pharmingen); Panel 2 = anti-CD11b-FITC (clone M1/70, BD Pharmingen), anti-CD11c-PE (clone HL3, BD Pharmingen), anti-Gr-1-AF700 (clone RB6-8C5, BD Pharmingen), and anti-F4/80-E450 (clone BM8, eBioscience); Panel 3 = anti-CD31-FITC (clone 390, BD Pharmingen) and anti-CD55-PE (clone RIKO-5, BD Pharmingen). Three antibody panels were assessed for cells from K/BxN treated mice as follows (clones as above): panel 1 = anti-CD4-FITC, anti-CD8-Pacific Blue and anti-CD19-PE; Panel 2 = anti-CD11b-FITC, anti-CD11c-PE, and anti-Gr-1-E450; Panel 3 = anti-CD31-FITC, anti-CD55-PE and anti-F4/80-E450. Cells were acquired using a FACSCanto I RUO analytical cytometer and analyzed using FACSDiva software (Becton Dickinson, San Jose, CA).

### Statistical analysis

Correlation was analyzed using Pearson Product Moment Correlation and Spearman Rank Order Correlation. Unpaired Students t-test was used to compare normally distributed parametric data between groups. Mann Whitney was used to compare nonparametric data between groups.

## Results

### SapC-DOPS localizes and accumulates for visualization of arthritic joints by fluorometric imaging in K/BxN and CIA arthritis models

**Figure 1:**
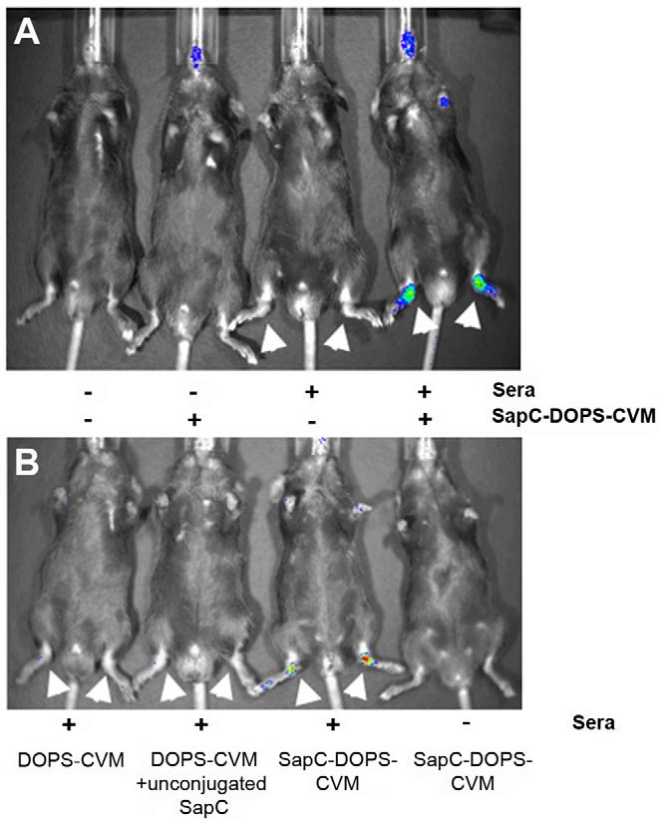
SapC-DOPS-CVM localizes to arthritic joints and is visible by live fluorometric imaging. Male C57Bl/6 mice three months of age were given 150 µl of sera i.p. as indicated. Seven days after sera injection, mice were imaged by IVIS® (A) five hours following SapC-DOPS-CVM i.v. injection as indicated (- is PBS); (B) two hours following i.v. injection of DOPS-CVM, DOPS-CVM plus uncoupled SapC, or SapC-DOPS-CVM as indicated. Arrows indicate macroscopically swollen paws.

To test the hypothesis that the PS targeting agent SapC-DOPS localizes to joint tissue following local destructive inflammatory events, the uptake of SapC-DOPS labeled with CVM (SapC-DOPS-CVM) was measured by optical imaging of mice challenged with two established models of inflammatory joint disease, each with distinct etiologies. First, mice challenged with K/BxN serum-transfer arthritis were examined seven days following sera injection. Mice were given SapC-DOPS-CVM and exhibited robust CVM fluorescence in macroscopically arthritic paws as compared to arthritic paws in mice not given SapC-DOPS-CVM (Figure 1A). Mice not treated with sera did not develop arthritis and correspondingly did not exhibit detectable CVM signal. In Figure 1B, mice challenged with K/BxN sera and receiving DOPS-CVM alone without SapC conjugation did not exhibit detectable signal, although arthritis was macroscopically apparent, demonstrating that SapC conjugation to DOPS-CVM is required for localization of CVM signal to arthritic paws. Administration of CVM alone also resulted in no detectable signal (data not shown).

**Figure 2:**
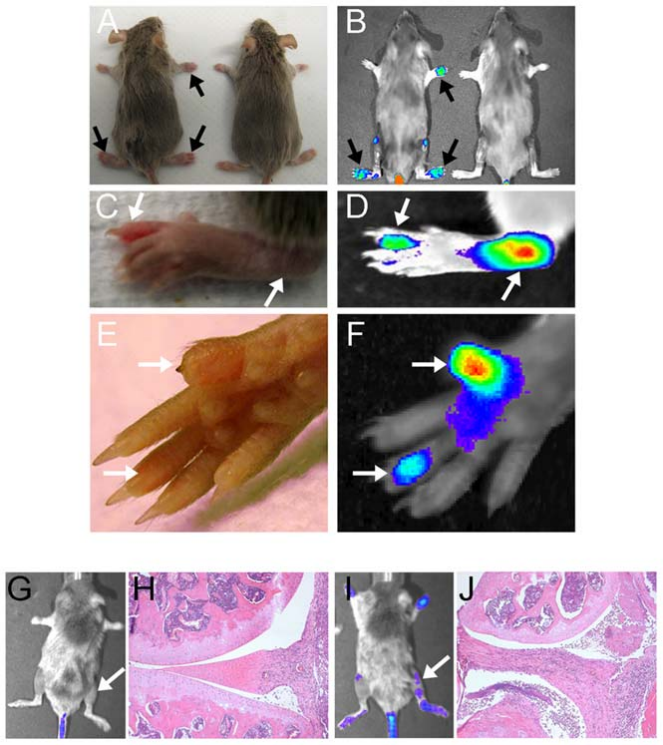
SapC-DOPS-CVM localizes to arthritic joints in CIA. (A) Light images of DBA/1 mice treated with CII (left, CIA) and non-CII treated (right, Control). (B) IVIS® images of mice in A, 48 hours following SapC-DOPS-CVM i.v injection. Light images of CIA hind paw (C) and forepaw (E) with corresponding IVIS® images in (D) and (F). Solid black arrows indicate macroscopically swollen paws and white arrows indicate macroscopically swollen joints. (G) and (I) IVIS® images of CII-treated mice 48 hours following SapC-DOPS-CVM i.v. injection, with arrows indicating corresponding histological knee sections (H) and (J) stained with hematoxylin and eosin. CVM intensity as measured by live fluorometric imaging is represented by red, yellow, green, blue colorimetric scale with red representing highest and blue representing lowest intensity.

SapC-DOPS-CVM targeting of arthritic tissue was further assessed by examining localization in mice challenged with CIA, one of the most well-characterized of the murine models of inflammatory joint disease. A cohort of DBA/1J mice was challenged, and as expected, macroscopic disease was apparent at 28 days following primary CII injection. Mice exhibiting macroscopically apparent arthritis also displayed localization of SapC-DOPS-CVM accumulation as determined by IVIS® imaging specifically in macroscopically arthritic, but not non-arthritic paws 28 days following primary CII injection (Figure 2). In mice that did not receive CII (Controls, Figures 2A and 2B) SapC-DOPS-CVM IVIS® signal was not detected. Furthermore, images focused on paws with arthritic digits (Figures 2C–2F) indicated that SapC-DOPS-CVM selectively accumulated to high levels in individual digits of a paw that were swollen, but not to those that were free of macroscopic evidence of disease, as shown by the swollen ankle and digit depicted in Figures 2C and 2D and in the forepaw digits shown in Figures 2E and 2F. Signal was also detected in knees (Figure 2B) from challenged mice as compared to control mice (no CII). While knee joints are not accessible for clinical scoring, further analysis of shaven mouse knees at day 28 following CII injection indicated that increased SapC-DOPS-CVM fluorescence was associated with arthritic knee joint histopathology. Figure 2G is representative of mouse knees that did not exhibit SapC-DOPS-CVM uptake (fluorescence) with little arthritic knee joint pathology (Figure 2H) including a small amount of infiltrating inflammatory cells and limited synovial hyperplasia. Figure 2I is representative of increased uptake of SapC-DOPS-CVM in mouse knees with marked arthritic pathology (Figure 2J) including abundant infiltration of inflammatory cells and synovial hyperplasia.

### SapC-DOPS targeting of arthritic joints over the course of disease correlates with disease severity

**Figure 3:**
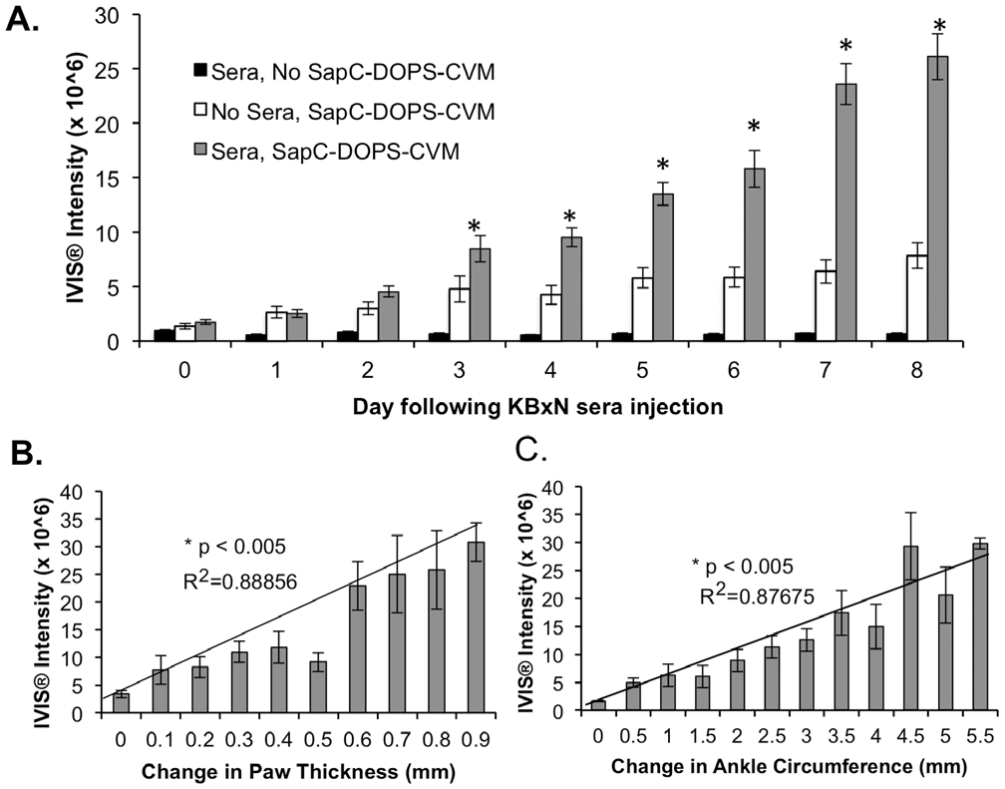
CVM signal intensity increases during the course of K/BxN arthritis and correlates with arthritic parameters. C57Bl/6 mice were given 150 µl of K/BxN sera i.p. and 84 µg of SapC-DOPS-CVM as indicated. (A) Means of back paw intensity values are graphed as columns with error bars representing standard error of the mean. * p<0.05, Students t-test, n≥4 mice per group. For mice treated with K/BxN sera (n = 7) and given SapC-DOPS-CVM the (B) changes in paw thickness and (C) changes in ankle circumference were rounded to the nearest millimeter and the means (+/− SEM) of IVIS® signal intensity for the corresponding paws are graphed as columns. Lines represent linear regression of values in B and C with correlation coefficients indicated. Correlation of arthritis parameters and IVIS® intensity signals in B and C is significant p<0.005, as determined by Pearson Product Moment Correlation and Spearman Rank Order Correlation.

Given that SapC-DOPS targets macroscopically apparent arthritic joints, it was of great interest to determine the kinetics of SapC-DOPS accumulation during the course of arthritic disease. Determination of SapC-DOPS-CVM localization over time provides information as to whether the intensity of signal correlates with the severity of disease. Since both the K/BxN and CIA models of inflammatory joint disease exhibit distinct etiologies and unique time courses of disease initiation and progression, SapC-DOPS uptake and accumulation was assessed over time in both models beginning with disease initiation and continuing through the peak of disease severity. First, K/BxN sera were administered to C57Bl/6 mice and animals were imaged by IVIS® six hours later and then daily. SapC-DOPS-CVM was administered every other day, beginning at the initial sera administration. Average radiance values (IVIS® intensity) of designated elliptical areas of the hind paws were determined and the mean values of groups for each day are indicated in Figure 3A. In K/BxN sera-challenged mice injected with SapC-DOPS-CVM, steady and significant increases were observed in intensity of CVM signal as shown in Figure 3A, with highest intensity at day 8 following K/BxN sera administration. As expected, control mice receiving K/BxN sera but no SapC-DOPS-CVM had low CVM signal (background radiance) that did not change throughout the analysis period despite robust arthritic disease development. Control mice receiving SapC-DOPS-CVM every other day but not K/BxN sera, showed a slow increase in fluorescent signal intensity, indicating that with multiple injections of agent a marginal background accumulation of the agent within the paws exists that quickly plateaued. Consistent with our hypothesis that SapC-DOPS-CVM targeting would correlate with disease severity, paws exhibiting higher positive changes in paw thickness (Figure 3B) and ankle circumference (Figure 3C) as measurements of arthritic severity concurrently exhibited higher levels of SapC-DOPS-CVM localization as indicated by IVIS® intensity. These data suggest that increases in arthritic severity as assessed by quantitative clinical arthritic measurements significantly correlate with increases in localization of SapC-DOPS to arthritic joints in the K/BxN model of arthritis.

**Figure 4:**
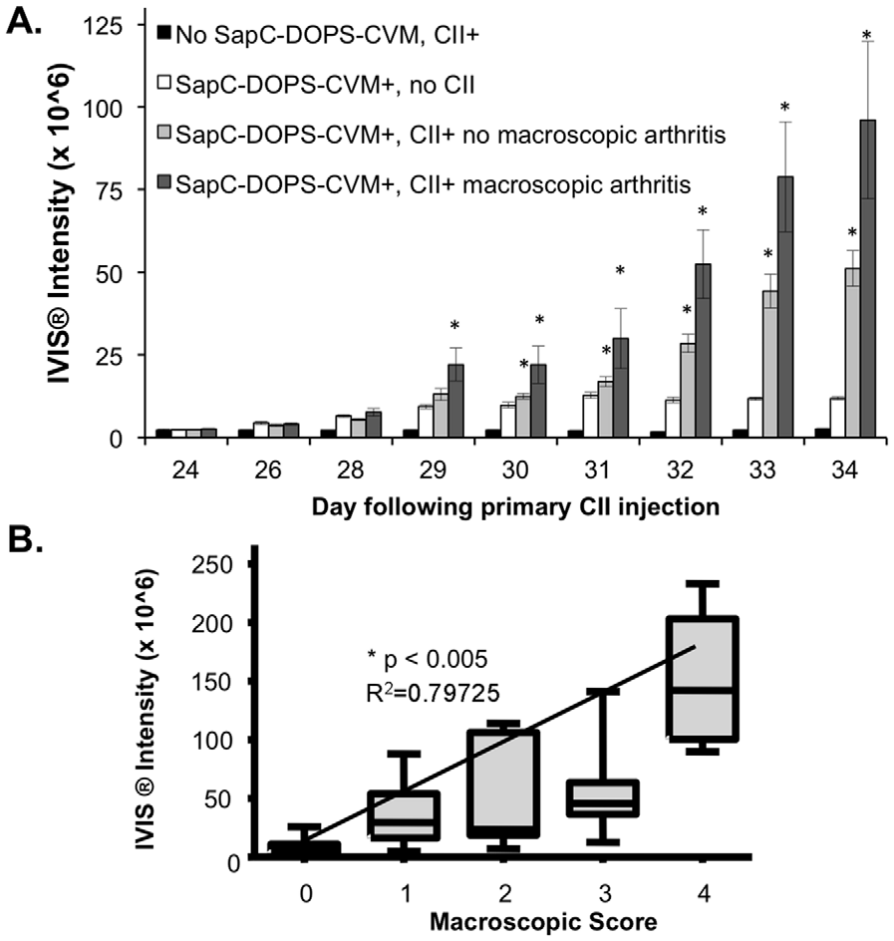
CVM signal intensity from CII-challenged mice increases over disease course and correlates with arthritic severity. (A) Mice were injected i.p. with SapC-DOPS-CVM starting on day 23 following primary CII immunization and then every other day. Mice were imaged daily by IVIS®. Columns indicate the mean IVIS® intensity values (average radiance) for paws in each group +/− the SEM. Mice were scored macroscopically for arthritis daily. N of paws imaged in groups were as follows: No SapC-DOPS-CVM, CII+: 16; SapC-DOPS-CVM+, no CII:16; SapC-DOPS-CVM+,CII+macroscopic arthritis: 8; SapC-DOPS-CVM+CII+ no macroscopic arthritis: 24. * = p<0.05 as compared to SapC-DOPS-CVM+, no CII, Mann Whitney (B) Box plots indicate the median values and range for IVIS® intensity of paws receiving the specified macroscopic arthritic score during the time course of disease. The line represents linear regression of mean values with the correlation coefficients indicated. Correlation of arthritis parameter values and IVIS® intensity signal values is significant, p<0.005, Pearson Product Moment Correlation and Spearman Rank Order Correlation.

In parallel analyses, CII-challenged mice were given SapC-DOPS-CVM at day 23 and every other day thereafter, and imaged daily from days 24–34 following CII primary immunization (Figure 4A). Similar to results in the K/BxN model, paws from mice immunized with CII and injected with SapC-DOPS-CVM showed prominent CVM signal at day 28 that steadily increased over the course of disease. Similar to the K/BxN arthritis experiments, the repetitive SapC-DOPS-CVM treatment regime resulted in low-level accumulation of SapC-DOPS-CVM signal in paws of mice receiving SapC-DOPS-CVM alone, without CII treatment, indicating background accumulation of the agent that was independent of disease pathogenesis. As expected fluorescent signal was not observed in paws of either control or arthritic mice that did not receive SapC-DOPS-CVM. Of note, paws from CIA-challenged mice, which received SapC-DOPS-CVM that were overtly macroscopically arthritic had the highest fluorescent intensity, and arthritic scores showed a positive correlation with increases in CVM intensity (Figure 4B). Perhaps an even more significant observation was that paws from CIA-challenged mice, which were macroscopically scored as non-arthritic, also showed significant increases in CVM fluorescence intensity as compared to control mice (not treated with CII) (Figure 4A). This observation suggests that SapC-DOPS-CVM may localize to joints with subclinical arthritis.

### SapC-DOPS-CVM is detectable in joint cell suspensions and accumulates primarily in CD11b+ Gr-1+ cells from K/BxN and CIA arthritic joints

**Figure 5:**
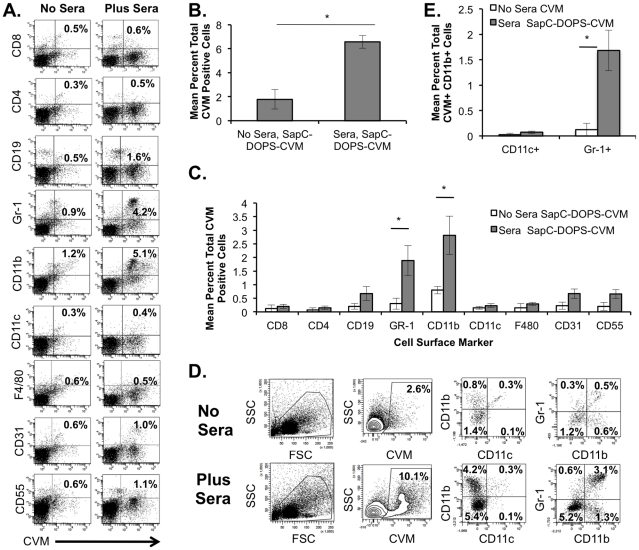
SapC -DOPS-CVM targets primarily CD11b+Gr-1+ joint cells in the K/BxN model of arthritis. Joint cells from hind paws were stained with antibodies to cell surface markers as indicated. FACS plots are representative from one animal, with bar graphs indicating the means +/− the SEM of groups. (A) Dot plots indicate cell populations that are CVM+ and positive for other cell surface markers as indicated. (B) CVM+ cells. Columns represent mean of total cells that are CVM+ +/− SEM with treatment of animals as indicated. * = p<0.05. (C) Bar graph indicates means +/− the SEM of the percentage of total cells that are CVM+ and positive for the indicated cell surface marker. * = p<0.05, Students t-test. (D) Dot plots show forward (FSC) and side scatter (SSC) for joint populations with gating for CVM+ cell populations versus side scatter (SSC). Gated CVM+ cells are further analyzed for specific cell-surface markers CD11b, CD11c and Gr-1 with percentages indicated. (E) Columns represent the mean +/− the SEM of the percentage of total cells that are positive for CVM, CD11b, and also positive for either CD11c or Gr-1 * = p<0.05. N = 4 No Sera, SapC-DOPS-CVM; N = 9 Sera, SapC-DOPS-CVM.

Since SapC-DOPS-CVM localizes specifically to arthritic joints, it was of great interest to determine cell types targeted by SapC-DOPS-CVM in both the K/BxN and CIA arthritis models. Cells extracted from joints were stained with labeled antibodies to cell-surface markers on lymphocytes (T cells: CD4, CD8; B cells: CD19), myeloid cells (CD11b, CD11c, Gr-1 and F4/80), endothelial cells (CD31), and synovial fibroblasts (CD55^high^) [18]. For K/BxN, at day nine following sera administration, mice were sacrificed and single cell suspensions were obtained from back paws of individual mice. Analysis of joint cells from mice treated with SapC-DOPS-CVM shows an increase in the CVM+ joint cells upon sera treatment (Figure 5A and 5B). Figure 5B indicates an average of ∼6–7% of the isolated live-gated joint cells were positive for CVM as opposed to only 1% of the cells staining positive for CVM in the no KBxN sera negative control. In Figure 5C the majority of cells to which CVM localizes also stained positively for the myeloid markers Gr-1 (mean = 1.9%, ranging from 0.2%–4.5%) and CD11b (mean = 2.8%, ranging from 1.1%–6.0%). Further analysis of the 6–7% SapC-DOPS-CVM positive cells for co-expression of cell surface markers indicates that approximately one-third express both CD11b and Gr-1 (mean = 1.7%, ranging from 0.5%–3.2) (Figure 5D and 5E), which is consistent with a neutrophil phenotype. None of the CVM+ cells stained positive for both Gr-1 and CD11c. Additionally, the expression of CD19, CD31 and CD55 in SapC-DOPS-CVM+ cells trended to be higher in joint cells from mice treated with K/BxN sera as compared to joint cells from the control mice (Figure 5C).

**Figure 6:**
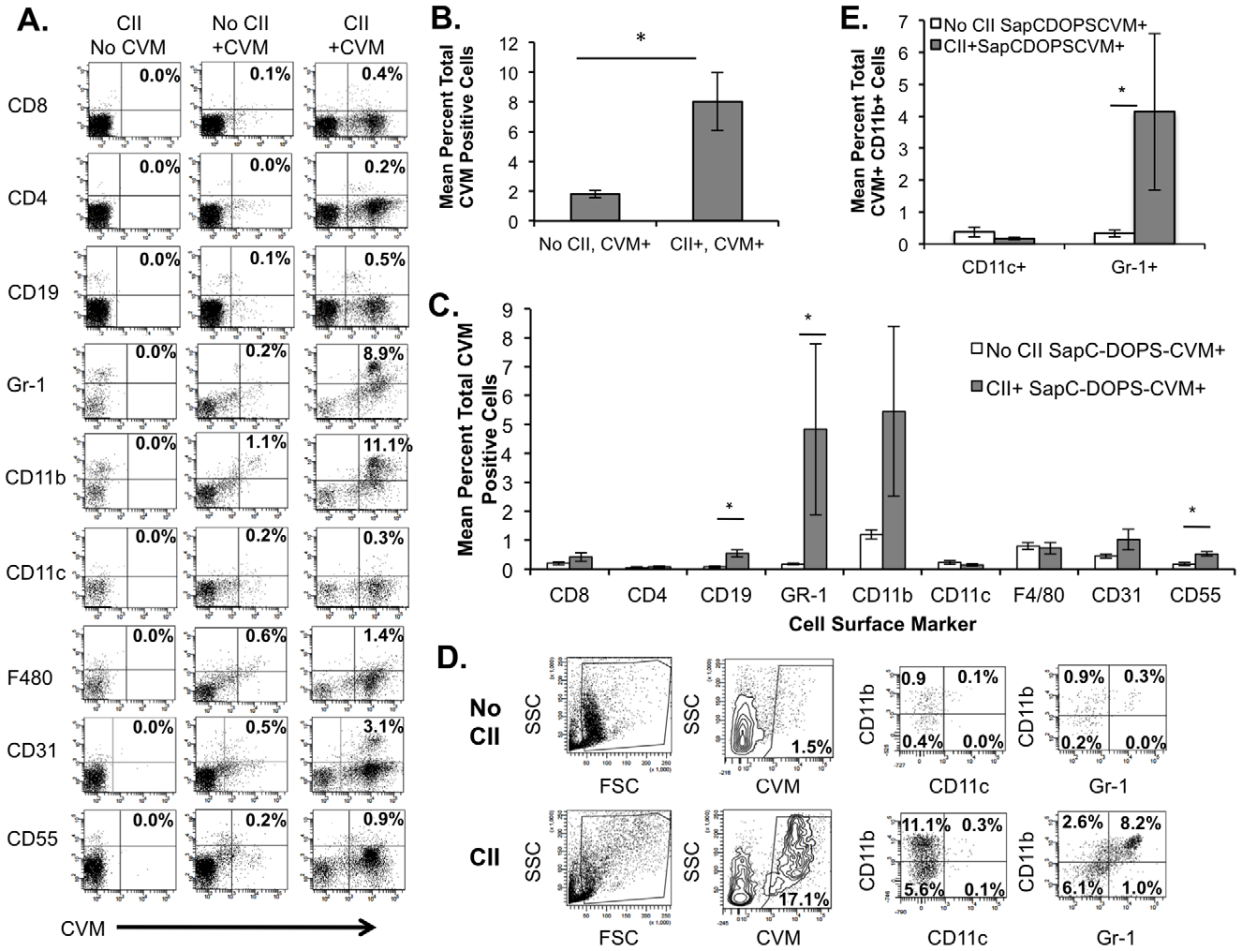
SapC -DOPS-CVM targets primarily CD11b+Gr-1+ joint cells in the CIA model of arthritis. Cells were stained with antibodies to cell surface markers as indicated. FACS plots are representative from one animal, with bar graphs indicating the means +/− the SEM of groups. (A) Dot plots indicate cell populations from mice treated as indicated that are CVM+ and positive for other cell surface markers as indicated on left. (B) CVM+ cells. Columns represent mean of total cells that are CVM+ +/− SEM (error bars) with treatment of animals as indicated. * p<0.0001 Mann Whitney. (C) Columns indicate means +/− the SEM (error bars) of the percentage of total cells that are CVM + and positive for the indicated cell surface marker. * = p<0.05 Mann Whitney. (D) Dot plots show forward (FSC) and side scatter (SSC) for joint populations with gating for CVM +cell populations versus side scatter (SSC). Gated CVM + cells are further analyzed for combinations of the specific cell-surface markers CD11b, CD11c and Gr-1 as indicated. (E) Columns represent the mean +/− the SEM (error bars) of the percentage of total cells that are positive for CVM, CD11b, and also positive for either CD11c or Gr-1. N = 4, No CII, SapC-DOPS-CVM; 8, CII+, SapC-DOPS-CVM+ * p<0.05, Mann Whitney.

For CIA, back paws were taken and joint cells pooled for analysis. Mice were sacrificed at 35 days following primary CII injection. Arthritic macroscopic scores were variable among the mice as would be expected for CIA with a median score of 1.5–2 for mice either given SapC-DOPS-CVM or not administered SapC-DOPS-CVM as a negative control. Disease incidence in the SapC-DOPS-CVM treated mice and the control non SAPC-DOPS-CVM treated animals was similar, with 63% (5/8 and 50% (4/8 affected) with overt macroscopic disease, respectively. Similar to the K/BxN mice, cells extracted from joints were stained with labeled antibodies for lymphoid, myeloid, endothelial and synovial fibroblast cells as described above. In Figure 6 joint cells from mice treated with SapC-DOPS-CVM show an increase in CVM+ cells upon CII challenge with the mean percent of CVM+ joint cells averaging approximately 8% of live cells (Figure 6B). CII-challenged mice showed populations that were positive for CD19, CD11b, CD55 and Gr-1, regardless of SapC-DOPS-CVM treatment by dot plot analysis (Figure 6A). However, the majority of CVM+ cells from CIA mice and given SapC-DOPS-CVM (CII, +CVM) were also positive primarily for CD11b (mean = 5.5%, range = 0.5% to 24.3%) and Gr-1 (mean = 4.8%, range 0.3% to 24.2%) (Figure 6C). As observed for the K/BxN joint cells, CVM+ populations also stained positive for CD19 (range 0.2% to 1.3%), CD31 (range 0.3% to 3.1%) or CD55 (range 0.3 to 0.9%). Figure 6D indicates the gating strategy for CVM+ populations, which were further analyzed for dual expression of specific cell surface markers. Similar to results with joint cells from K/BxN-challenged mice, a significant portion (mean = 5.4%, ranging from 0.3% to 20%) of the SapC-DOPS-CVM+ cells also expressed both CD11b and Gr-1 on their cell surface, but not CD11c (Figure 6E) and CVM+ cells did not stain positive for both Gr-1 and CD11c. The variability in SapC-DOPS-CVM+ cell populations extracted from individual mice is consistent with the variability in the penetrance of disease as cells from both hind paws were pooled for cell analysis.

## Discussion

The present studies investigate the utility and underlying mechanism(s) of SapC-DOPS nanovesicles as an optical imaging agent to detect tissue damage in inflammatory arthritic joints. In two separate animal models of inflammatory arthritis, SapC-DOPS labeled with the fluorophore CVM was visualized locally in arthritic inflammatory joints. Furthermore, throughout the course of both K/BxN and CIA inflammatory arthritis SapC-DOPS intensity increased and correlated with increases in multiple independent parameters of arthritic disease assessment. Joint cells targeted by SapC-DOPS-CVM were primarily positive for both CD11b and Gr-1—cell markers indicative of neutrophils—that have previously been shown to be critical mediators of inflammatory events in arthritis [19]–[22]. The use of SapC-DOPS to target imaging agents to areas of joint inflammation provides a potentially novel, noninvasive, and robust tool in the assessment of inflammatory arthritis.

Optical imaging has great promise for use in inflammatory arthritis, particularly in RA, in which the peripheral joints are the most highly affected. Assessment of joint damage in RA has been measured primarily through x-ray imaging analysis, which does not readily provide information regarding the earliest local pathogenic events (i.e., inflammation), which occur prior to joint damage. MRI and ultrasound are beginning to be used as modalities to assess soft tissue damage; however, each of these modalities has significant factors hindering their routine use in the clinic. Optical imaging in humans has primarily involved imaging of vasculature and lymphatics using the non-specific, near-infrared dye indocyanine green [23], with more recent advances using fluorescent-labeled folate receptor-alpha targets for tumor-specific, fluorescence-assisted cytoreductive surgery [24]. For RA patients, visualization of indocyanine green uptake focusing on the finger and wrist joints has been demonstrated with newly developed optical imaging instrumentation [25]. In animal models of arthritis, anti-E-selectin labeled with a near-infrared fluorophore demonstrated specificity for arthritic joints in CIA mice [26]. In addition, antibody and folate receptor targets have been used to image activated macrophages in arthritis models and other inflammatory settings [27]–[29].

Using optical imaging our studies indicate that SapC-DOPS-CVM appears to localize to arthritic joints. Our prior optical imaging studies regarding tissue-specific distribution of SapC-DOPS-CVM signal demonstrated fluorescent signal in only liver and spleen of otherwise unchallenged nude mice that diminished over time and was not detectable 48 hours following injection [16]. Our present data indicate that in two separate models of arthritis, SapC-DOPS-CVM is detected by optical imaging in arthritic joints. Similar results were obtained in a third model of arthritis in which chronic polyarthritis is driven solely by overexpression of human TNF-alpha via a transgene [30]. SapC-DOPS-CVM was clearly localized to arthritic joints of TNF-alpha transgenic mice but not to joints of littermate control transgene negative animals (data not shown). Thus, this novel compound appears to selectively target arthritic tissues, regardless of disease etiology, giving it a monumental advantage over imaging through the use of non-specific fluorophores.

SapC-DOPS-CVM localization as assessed by optical imaging may be useful as a marker for both the detection of early pre-clinical disease and for closely monitoring disease progression. Analysis of SapC-DOPS-CVM accumulation in joints over the course of both K/BxN and CIA arthritis indicated an increase in CVM signal as arthritis progressed. A significant finding of the present study is that clinical parameters, such as paw thickness and ankle circumference in the K/BxN model showed significant correlations with CVM intensity (i.e., increased severity of disease correlated with higher signal intensity). Non-sera treated mice that received SapC-DOPS-CVM every other day, accumulated significant low-level signal that plateaued early in disease. This result indicates that such low level of fluorescent signal may accumulate non-specifically due to the repeated administration of the fluorescent agent but would not hinder detection of early arthritic disease. While our studies focus on proof-of-principle for assessing joint damage in a mouse model over time, in clinical practice low level signal accumulation would not be expected since multiple injections over a short period of time would not be necessary. In parallel CIA analysis, signal intensity of SapC-DOPS-CVM localization to paw joints also increased in CII-challenged mice. In CII-treated mice, macroscopically arthritic paws showed a significant correlation with increased CVM signal intensity. Interestingly and specifically pertinent to early detection of inflammation and disease, in CII challenged mice a limited number of paw joints that were not overtly arthritic showed an increase in CVM signal intensity, which we believe may represent the earliest stages of arthritis. Future studies will histologically assess microscopic arthritis over the time course of disease to directly assess the histological changes that might be occurring sub-clinically in these paws as related to SapC-DOPS-CVM accumulation. Our results did not indicate any affects on arthritic disease progression and severity as both K/BxN and CIA arthritic disease progressed similarly to previous experiments performed in our laboratory; however, the results of the current study do not absolutely exclude the possibility that SapC-DOPS alone alters the kinetics and severity of arthritic disease.

Analysis of SapC-DOPS-CVM targeted cells extracted from arthritic joints indicates that the main cell type targeted is CD11b and Gr-1 positive. Mature granulocytes, particularly neutrophils that reside in tissues, express Gr-1 on their cell surface [31], [32]. Neutrophils are short-lived cells that are involved in innate immunity and host defense but have also been shown to be involved in mechanisms that drive RA [19]–[22], [33]. Deficits in the ability of neutrophils to undergo apoptosis have been associated with autoimmune disorders including RA [34]. Disruptions in neutrophil activation mechanisms (i.e., degranulation, oxidative burst) have been shown to limit the local tissue damage commonly associated with inflammatory joint disease [35], [36]. Neutrophil activation with production of reactive oxygen species leads to phospholipid oxidation and subsequent externalization of PS [37]. Neutrophils are one of the most abundant cell types in synovial fluid from RA patients and products of neutrophil activation are present in RA synovial fluid as well [38]. Thus, SapC-DOPS targeting of this cell type is not surprising, as activated neutrophils readily externalize PS. Furthermore, the resistance of neutrophils to apoptosis in RA, potentiates the usefulness of SapC-DOPS binding as a clinical measure. A large portion of targeted cells did not stain positive for CD11b or Gr-1, indicating that other cell types are also targeted by SapC-DOPS-CVM, and future studies with more extensive cell markers will reveal additional targeted cell types. However, other cell types targeted by SapC-DOPS-CVM within our studies include CD19+ cells, which is a B-cell marker; CD31, a marker for endothelial cells; and CD55, a marker that is expressed at high levels on synovial fibroblasts [18]. All of these cells are involved in the early inflammatory stages of arthritic disease, providing further support for use of this agent in early detection of inflammatory joint disease [39]–[46].

A significant finding from these studies was that SapC-DOPS is able to target arthritic joints in multiple disease models that exhibit distinct etiologies [47], [48]. The targeting of SapC-DOPS correlated directly to the course and pathogenesis of disease in both arthritis models. In K/BxN mice 100% penetrance of hind paws is normally observed by day 8 of disease. Our data indicated a clear targeting of SapC-DOPS-CVM to hind paws in K/BxN arthritis over the time course of disease and a direct correlation with paw thickness and ankle circumference. In addition, joint cells taken at day 8 of disease showed SapC-DOPS targeting of CD11b+Gr-1+ cells, which is consistent with a neutrophil phenotype. Neutrophils have been implicated as a major driver of pathogenesis in the K/BxN arthritis model [40], [49]. For CIA, disease incidence is slightly less with ∼50–100% of mice developing overt macroscopic disease with both distal (i.e., paw joints) and proximal (i.e., knees) joint involvement that is not uniform, similar to the sporadic joint involvement exhibited by RA patients. In contrast with the K/BxN model in which disease appears quickly, macroscopic signs of arthritis in CIA can become apparent weeks following the secondary collagen immunization [50]. In addition, the higher degree of variability observed in the analysis of cell types targeted by SapC-DOPS in the CIA model are likely reflective of both the variability and time course of joint involvement in the CIA model. Given the more complex disease process of CIA [46], SapC-DOPS is likely targeting multiple cell types involved in the pathogenesis of arthritis. Nevertheless, the significant correlation between SapC-DOPS-CVM intensity and arthritic severity observed in the both the K/BxN and CIA models suggests that common pathological mechanisms (i.e., the influx of myeloid derived cells of the innate immune system) exist that are being targeted by SapC-DOPS. In total, our observations strongly suggest that targeting arthritic joints using SapC-DOPS may have far-reaching applicability in a variety of arthritic contexts.

The striking observation on arthritis-selective targeting of the novel compound SapC-DOPS suggests that this molecule may be used to detect inflammation in a variety of settings and contexts. Studies directed at evaluating whether SapC-DOPS can detect subclinical inflammation; other arthritides; and/or other inflammatory disorders remain to be performed; however, the results presented here argue that SapC-DOPS facilitated imaging may be used as a clinical parameter that could be highly valuable to assess inflammatory joint disease. One of the advantages to SapC-DOPS is its flexibility in incorporating other agents within the nanovesicles and targeting inflammatory regions. This technology also has the promising potential for specific delivery of agents to joints, which would be beneficial particularly with respect to therapies that when administered globally have undesirable side effects.
